# Concordance of intraoperative cytology with frozen section and final pathology for ovarian neoplasms^☆^

**DOI:** 10.1016/j.gore.2025.101757

**Published:** 2025-04-29

**Authors:** Jacqueline Early, Gagandeep Kaur, Lisa Arrigo, Skylar Gill, Mary Chacho, Paul Fiedler, Linus Chuang

**Affiliations:** a Department of Obstetrics and Gynecology, Danbury Hospital, Danbury, CT, USA; b Rudy L. Ruggles Biomedical Research Institute, Danbury, CT, USA; c Department of Pathology and Laboratory Medicine, Danbury Hospital, Danbury, CT, USA

**Keywords:** Intraoperative frozen section, Ovarian mass, Intraoperative cytology

## Abstract

**Introduction:**

Intraoperative frozen section analysis (IOFS) is a widely used method for evaluating ovarian masses and guiding surgical decisions. However, IOFS is not always accessible in low-resource settings. Recent research suggests that intraoperative cytology (IOC) may offer a quicker, less equipment-dependent alternative. This study aims to compare IOC and IOFS in women with ovarian neoplasms. The pathologists interpreting IOC were blinded to IOFS findings.

**Methods:**

The study included 15 women aged 18 or older undergoing surgery for ovarian mass removal. Patients with biopsy-confirmed malignancies or known recurrences were excluded. After tumor removal, both IOFS and IOC were performed. IOFS involved using a Cryostat for sectioning and H&E staining. IOC used touch/imprint, scrape, and crush techniques. Independent pathologists analyzed the smears, and paraffin-embedded tissue histopathology was the diagnostic gold standard.

**Results:**

IOC results were consistent with final pathology in 10 of 15 cases, while IOFS matched in 12 cases. IOC had 4 indeterminate and 1 borderline diagnosis, compared to 1 indeterminate, 1 benign, and 1 borderline diagnosis with IOFS. Three discrepancies between IOC and IOFS were identified, with IOC providing indeterminate results where IOFS ranged from benign to malignant. Indeterminate cases led to malignancy management strategies, including intraoperative staging.

**Conclusion:**

This pilot study indicates that intraoperative cytology is a practical and effective method for rapid ovarian neoplasm evaluation. Further research with larger cohorts is needed to assess its comparative performance with frozen section analysis and optimize its methodology. IOC may offer a viable option for intraoperative surgical guidance.

## Highlights

• Frozen section, with sensitivity and specificity, offers a preliminary diagnosis.
• Intraoperative cytology can provide faster results with minimal specialized equipment.
• Preliminary data suggests IOC is a viable replacement for when IOFS is unavailable.
• IOC should be studied as an effective tool for evaluating ovarian neoplasms.

## 1 Introduction

Intraoperative evaluation with frozen section pathologic assessment of an ovarian mass has become a standard of care for treatment of these lesions. Traditionally, the ovarian tumors are not biopsied prior to surgery due to the risk for intraperitoneal seeding of a possible malignancy. Therefore, these lesions are not usually known as benign or cancerous before surgery and the recommendations for surgical management differ based on the benign or malignant pathology.

There are several options for management, including universal staging procedures where every patient receives full cancer staging, and the use of delayed staging after an initial diagnostic surgery. The first has a significant risk of over-treatment, as staging may include bilateral salpingoophorectomy, total hysterectomy, pelvic and paraaortic lymph node dissections and omentectomy. These additional procedures in addition to removal of an ovarian mass are associated with increased morbidity and mortality (Abu-Rustum et al., 2006). The second option carries a significant risk and strain on the patient physically, emotionally and financially. The common practice has been to evaluate the tumors using frozen section, a preliminary histopathologic diagnostic tool to assist in intraoperative decision making.

Frozen section has been well established in the literature on ovarian neoplasms as an intraoperative assessment of malignancy, with a sensitivity and specificity of 90.0 % and specificity at 99.5 % (Ratnavelu et al., 2016), for differentiation of a mass into benign, borderline and malignant processes. This analysis has been compared to a gold standard of histopathologic diagnosis on paraffin section, which on average takes a week to process. Due to its high sensitivity and specificity, frozen section has been used as an intraoperative adjunct for surgeon’s decision making to help patients avoid the risks from unnecessary surgery and complete staging in one session. However, like any test it has limitations. Increased time in the OR increases the risk to the patient and increases costs. The frozen section on average takes 27 min to process (Esbona et al., 2012). Additionally, a frozen section requires specialized equipment and can often be unavailable at locations with fewer resources. More recent literature has described intraoperative cytology assessment as comparable to frozen section, with similar sensitivity and specificity but with faster turnaround times and reduced need for specialized equipment (Mat Zin and Zulkarnain, 2019).

Several methods of intraoperative cytology are available, with quick and accurate pathologic analysis of tissue specimens. Lesions can be subdivided into benign, borderline and malignant neoplasms to guide surgical management, as with frozen section. Intraoperative cytology on average takes less time to process, on average 13 min (Esbona et al., 2012). Multiple methods of cytology are available, including touch/imprint cytology, scrape cytology and crush cytology. These cytology methods have been shown to have excellent preservation of the cellular details of the specimen, with minimal artifact as evidenced in frozen section. Despite these advantages, IOC adoption still needs to be improved. One contributing factor is the lack of robust comparative data evaluating IOC against IOFS and final histopathology, particularly for ovarian neoplasms.

This study aims to address this gap by systematically comparing the diagnostic accuracy of IOC and IOFS with the gold standard of final paraffin histopathology. By evaluating concordance rates, diagnostic discrepancies, and the clinical implications of indeterminate cases, this research seeks to determine whether IOC can serve as a viable substitute for IOFS. The findings are particularly relevant for resource-limited environments, where the absence of IOFS often compromises intraoperative decision-making. This preliminary investigation provides critical insights into IOC’s feasibility, cost-effectiveness, and reliability, potentially broadening its role in surgical oncology.

## 2 Methods and materials

This prospective pilot study was conducted in a U.S.-based hospital but designed to simulate low-resource contexts by evaluating IOC as an alternative in resource-constrained settings. It evaluated the diagnostic concordance of intraoperative cytology (IOC) and intraoperative frozen section (IOFS) with final histopathology in women undergoing surgery for ovarian mass removal. Fifteen patients aged 18 years or older were prospectively enrolled, and informed consent included explanation of IOC and IOFS use and potential influence on intraoperative surgical decisions. Patients with biopsy-confirmed malignancies or known recurrences were excluded to ensure that the study population consisted of cases requiring intraoperative evaluation for diagnostic and management purposes. This inclusion criterion allowed for assessing IOC and IOFS in situations where their diagnostic utility was most relevant. The study provided a realistic evaluation of these tools in clinical scenarios that demand real-time decision-making.

After routine removal of the ovarian mass, the specimens were subjected to both IOFS and IOC in parallel. The IOFS was conducted using Cryostat machines (Leica CM1900 at two locations) set between − 25° and − 30 °C to produce 5 μm sections. They were then stained with hematoxylin and eosin (H&E) stain for histopathological evaluation. IOFS served as the interoperative standard for comparison. While there is no universally defined optimal amount, at least three representative cytologic smears (touch, scrape, crush) were prepared for each case. IOC was performed using three cytologic preparation methods: An imprint cytology was performed by pressing the cut surface of the specimen to a glass slide. Scrape cytology was performed by scraping the specimen gently with a scalpel and smearing the material to a slide. A crush cytology was performed by compressing tissue samples between slides and examining the resulting smear. Each slide was subsequently stained with hematoxylin and eosin for analysis. Final pathology will be determined by histopathology on permanent sections of paraffin-embedded tissue, which has been fixed in 10 % buffered formalin. Histopathologic diagnosis using paraffin-embedded tissue was considered the gold standard for statistically evaluating cytologic diagnosis.

Pathologists were blinded to IOFS and final histopathology results, and they independently interpreted the IOC findings. This methodological design ensured unbiased evaluation of the cytologic preparations. We compared IOC and IOFS results using concordance versus discordance with final histopathology as the reference standard. These data were secondarily compared to the final paraffin section. This approach allowed for a robust comparison of the accuracy and utility of IOC and IOFS in real-world surgical settings.

The primary outcome measure was the diagnostic concordance rate of IOC and IOFS with final histopathology. Concordance was defined as agreement between the intraoperative diagnosis (IOC or IOFS) and the final histopathological diagnosis. Secondary analyses focused on the rate of indeterminate diagnoses and their impact on intraoperative management decisions. Indeterminate cases, often the most challenging for surgeons, were analyzed to assess their influence on the surgical course and their implications for patient care.

## 3 Results

### 3.1 Concordance rates

The diagnostic performance of intraoperative cytology (IOC) and intraoperative frozen section (IOFS) was assessed by comparing their interpretations with the final histopathologic diagnosis. Among the 15 cases included in the study, IOC findings were concordant with final pathology in 10 cases (66.6 %), whereas IOFS demonstrated concordance in 12 cases (80 %) Table 1.

**Table 1:** Comparison of IOC and IOFS to final histopathologic diagnosis.

Patient #	IOC	IOFS	Final Diagnosis
1	Benign	Benign	Benign
2	Borderline	Borderline	Malignant
3	Benign	Benign	Benign
4	Benign	Benign	Benign
5	Benign	Benign	Benign
6	Benign	Benign	Benign
7	Malignant	Malignant	Malignant
8	Benign	Benign	Benign
9	*Indeterminant*	*Benign*	*Malignant*
10	*Indeterminant*	*Indeterminant*	*Malignant*
11	Malignant	Malignant	Malignant
12	*Indeterminant*	*Malignant*	*Malignant*
13	Malignant	Malignant	Malignant
14	*Indeterminant*	*Benign*	*Benign*
15	Malignant	Malignant	Malignant

IOC yielded four indeterminate diagnoses and one borderline interpretation. In contrast, IOFS resulted in one indeterminate diagnosis, one case misclassified as benign, and one borderline diagnosis that did not match the final pathology. These results suggest that IOFS had a higher overall concordance rate with final diagnosis, though both methods exhibited some diagnostic limitations.

Three cases demonstrated notable discrepancies between IOC and IOFS. In Case #9, IOC classified the lesion as indeterminate, while IOFS diagnosed it as benign. However, final histopathology revealed malignancy, underscoring a false-negative result from IOFS. In Case #12, IOC again labeled the sample as indeterminate, but IOFS accurately diagnosed malignancy, which was confirmed by final pathology. Finally, in Case #14, IOC was indeterminate, IOFS diagnosed the lesion as benign, and the final diagnosis confirmed benign disease Table 2.

**Table 2:** Comparison of preoperative imaging characteristics, ovarian mass sizes, and CA-125 levels, IOC and IOFS to final histopathologic diagnosis.

Patient #	Preoperative imaging characteristics (ORADS)	Tumor size in greatest dimensions (cm)	Tumor marker (CA 125 U/mL)	IOC	IOFS	Final Diagnosis
1	5	16	153.1	Benign	Benign	Benign
2	5	21	27	Borderline	Borderline	Malignant
3	4	10.8	12	Benign	Benign	Benign
4	3	Bilateral 5.7	13	Benign	Benign	Benign
5	5	26	29	Benign	Benign	Benign
6	4	4.8	30	Benign	Benign	Benign
7	5	5.1	178	Malignant	Malignant	Malignant
8	1	5	6	Benign	Benign	Benign
9	*5*	*5*	*59*	*Indeterminant*	*Benign*	*Malignant*
10	*5*	*18*	*3768*	*Indeterminant*	*Indeterminant*	*Malignant*
11	5	24	1106	Malignant	Malignant	Malignant
12	*5*	*Bilateral 4.6 cm*	*636*	*Indeterminant*	*Malignant*	*Malignant*
13	5	Bilateral 13.7	35	Malignant	Malignant	Malignant
14	*4*	*4*	*15*	*Indeterminant*	*Benign*	*Benign*
15	4	Bilateral 3.9	284	Malignant	Malignant	Malignant

These cases highlight the inherent challenges in intraoperative pathology interpretation, particularly when attempting to classify borderline or indeterminate tumors. Despite the higher rate of indeterminate results, IOC appeared to prompt a more cautious intraoperative approach, as surgeons proceeded with malignancy staging in those instances, potentially avoiding the need for a second surgery.

### 3.2 Clinical implications

In all cases where IOC provided indeterminate results, surgeons performed malignancy staging based on the precautionary principle. Staging decisions were precautionary when IOC is indeterminate, not definitive; the final staging relied on the gold standard diagnosis. This approach aimed to avoid the need for repeat surgeries while ensuring timely and comprehensive care. The study findings underscore the potential of IOC to guide intraoperative management effectively, even in cases where its diagnostic precision is limited.

## 4 Discussion

### 4.1 The role of intraoperative cytology as a diagnostic Tool: Advantages and limitations

The findings of this study underscore the potential of intraoperative cytology (IOC) as a complementary or alternative diagnostic tool to intraoperative frozen section (IOFS) inevaluating ovarian neoplasms. While IOC demonstrates slightly lower diagnostic accuracy than IOFS, its unique logistical advantages make it a particularly valuable resource in settings with limited access to specialized equipment or trained personnel, with overall lower costs (Esbona et al., 2012, Tanaka et al., 2020, Bhardwaj et al., 2019, Bohara et al., 2018, Mat Zin and Zulkarnain, 2019 Feb 26, Zulkarnain et al., 2020 Oct 1, Rao et al., 2009, Khunamornpong and Siriaunkgul, 2003, Shahid et al., 2012). These advantages and its ability to deliver rapid and reliable results position IOC as a promising option for guiding intraoperative decision-making.

### 4.2 Advantages of intraoperative cytology

One of the most significant benefits of IOC is its faster turnaround time. On average, IOC provides results in approximately 13 min, compared to 27 min for IOFS (Esbona et al., 2012). This efficiency reduces the time a patient spends under anesthesia, potentially decreasing overall surgical risk and improving operating room efficiency. This expedited process is particularly beneficial in high-volume surgical settings or emergencies where quick decision-making is essential.

Another notable advantage of IOC is its ability to preserve nuclear and cytoplasmic details with remarkable clarity (Kumar Verma et al., 2013). Unlike IOFS, which can introduce artifacts such as ice crystal formation during tissue freezing (Mat Zin and Zulkarnain, 2019), particularly among softer specimens (Mat Zin and Zulkarnain, 2019 Feb 26, Nanarng et al., 2015), IOC employs simple preparation methods like touch/imprint, scrape, and crush cytology. These techniques produce high-quality smears with minimal distortion, allowing pathologists to analyze cellular morphology effectively. This capability is especially important for identifying specific features of neoplastic cells, such as nuclear atypia or mitotic activity, which are critical for distinguishing benign, borderline, and malignant conditions.

IOC is also highly applicable in scenarios where tissue availability is limited. The cytology techniques used in IOC require only small samples, preserving the integrity of the remaining tissue for further diagnostic evaluation or research (Mat Zin and Zulkarnain, 2019). This attribute is particularly advantageous in cases where tissue biopsies must be conserved for subsequent molecular or genetic studies.

### 4.3 Challenges and limitations of intraoperative cytology

Despite its many benefits, IOC has challenges. One of the most significant limitations is its higher rate of indeterminate diagnoses than IOFS. Indeterminate results can create uncertainty in surgical planning, potentially leading to overtreatment or delayed definitive management. In this study, IOC classified more cases as indeterminate than IOFS, highlighting the need to refine cytology techniques and interpretative criteria further to improve diagnostic precision.

The quality of IOC results also heavily depends on the operator's skill and experience in preparing the slides. Adequate cellular yield and minimal artifacts require careful specimen handling, and inconsistent preparation techniques can compromise diagnostic accuracy (Rao et al., 2009, Mat Zin and Zulkarnain, 2019 Feb 26). Training programs and standardized protocols are essential to address this operator dependency and ensure the reproducibility of IOC findings across different institutions.

Another limitation of IOC is its restricted ability to assess complex tissue architecture.

While IOC excels at evaluating individual cells, it lacks the spatial context IOFS provides, allowing pathologists to examine tissue patterns and relationships. This limitation may hinder the detection of certain borderline or multifocal neoplasms that rely on architectural features for diagnosis.

## 5 Conclusion

Intraoperative cytology offers a viable alternative to frozen section analysis, particularly in resource-constrained settings where speed, simplicity, and minimal equipment requirements are paramount. Its faster turnaround time, comparable preservation of cellular details, and adaptability to limited tissue scenarios make it a powerful diagnostic tool. However, the higher rates of indeterminate diagnoses, operator dependency, and limited architectural assessment highlight areas for improvement and further research. With advancements in training, techniques, and interpretative criteria, IOC has the potential to play an increasingly prominent role in intraoperative diagnostics, ensuring timely and effective surgical management in diverse healthcare settings. This is a feasibility pilot study and further research with larger cohorts is needed prior to broader adoption.

## Funding statement

The authors received no funding for the work discussed in this manuscript.

## CRediT authorship contribution statement

**Jacqueline Early:** Writing – review & editing, Writing – original draft, Visualization, Validation, Formal analysis. **Gagandeep Kaur:** Investigation, Conceptualization. **Lisa Arrigo:** Writing – review & editing, Visualization, Project administration, Investigation, Formal analysis, Data curation, Conceptualization. **Skylar Gill:** Writing – review & editing, Visualization, Validation. **Mary Chacho:** Methodology, Conceptualization. **Paul Fiedler:** Writing – review & editing, Visualization, Validation, Methodology, Conceptualization. **Linus Chuang:** Writing – review & editing, Visualization, Validation, Methodology, Formal analysis, Conceptualization.

## Declaration of competing interest

The authors declare that they have no known competing financial interests or personal relationships that could have appeared to influence the work reported in this paper.

## Data Availability

Data will be made available on request.
